# Acceptance of Provider Initiated HIV Testing and Counseling among Tuberculosis Patients in East Wollega Administrative Zone, Oromia Regional State, Western Ethiopia

**DOI:** 10.1155/2014/935713

**Published:** 2014-03-20

**Authors:** Wakjira Kebede, Fikru Keno, Temesgen Ewunetu, Gutu Mamo

**Affiliations:** ^1^Department of Medical Laboratory Sciences and Pathology, College of Public Health and Medical Sciences, Jimma University, Jimma, Ethiopia; ^2^Oromia Regional Social and Labor Affairs Agency, Addis Ababa, Ethiopia; ^3^Department of Pharmacy, College of Public Health and Medical Sciences, Nekemte Hospital, Jimma, Ethiopia

## Abstract

Human immunodeficiency virus (HIV) is a powerful risk factor for the development of tuberculosis. This study assessed the acceptance and associated factors that can affect provider initiated HIV testing and counseling (PITC) among tuberculosis patients at the East Wollega administrative zone, Oromia regional state, western Ethiopia, from January to August, 2010. A single population proportion formula is used to calculate the total sample size of 406 and the cluster sampling technique was used to select 13 health centers that provide PITC services. The sample size was proportionally allocated to each health center. The study participants were selected using a simple random sampling technique using the lottery method. Structured questionnaire was used for collection of sociodemographic data. From the total of study subjects, 399 (98.2%) TB patients were initiated for HIV test and 369 (92.5%) patients accepted the initiation. Of those, 353 (95.5%) patients had taken HIV test and received their results. According to the reviewed documents, the prevalence of HIV among tuberculosis (TB) patients in the study area was 137 (33.7%). The logistic regression result showed the PITC was significantly associated with their knowledge about HIV (AOR = 3.22, 95% CI: 1.3–7.97), self-perceived risk (AOR = 2.93, 95% CI: 1.12–7.66), educational status (AOR = 3.51, 95% CI: 1.13–10.91), and knowledge on transmission of HIV/AIDS (AOR = 7.56, 95% CI: 1.14–40.35) which were significantly associated with the acceptance of PITC among TB patients. Therefore, this study's results showed, the prevalence of HIV among TB patient was high; to enhance the acceptance of PITC among TB patients, health extension workers must provide health education during home-to-home visiting. TB treatment supervisors also provide counseling intensively for all forms of TB patients during their first clinical encounter.

## 1. Introduction

World Health Organization (WHO) estimates that one-third of the world's population is infected with* Mycobacterium tuberculosis*, resulting in an estimated nearly 8.7 million incident cases of tuberculosis (TB) and 1.4 million deaths in 2012. TB has been closely linked to human immunodeficiency virus (HIV) and is believed to account for 50–80% in parts of sub-Saharan Africa for 500,000 deaths; 25% of the total deaths occur in people living with HIV [1–3]. Worldwide, TB is the most common opportunistic infection affecting HIV-seropositive and it remains the most common cause of death and major threat in terms of its personal, family, and socioeconomic consequences [4].

WHO Global report of 2012 ranked Ethiopia as the seventh among the highest TB burden countries in the world, with an estimated incidence and prevalence of all forms of TB cases being 258, 237/100,000 population, respectively [5]. Ethiopia Ministry of Health Hospital statistics data show that TB is the third leading cause of outpatient morbidity and mortality and the fourth leading cause of hospital admission [6].

HIV is the main reason for failure to meet TB control targets in high HIV settings, because HIV infection has contributed to progressive decline in immune response and pathogenesis of TB, increasing the risk of coinfection leading to more frequent extrapulmonary disease [4, 7]. The risk of reactivating TB infection in HIV infected persons is 7–10% per year compared with 10% lifetime risk in those serum negative persons. In order to control TB in high HIV settings, the Stop TB strategy needs collaborative TB/HIV activities in the country [4, 5, 8].

Worldwide only 20% of people living with HIV know their status, and only one in four people estimated to be living with HIV and TB is detected and treated for both diseases [3]. In sub-Saharan Africa only 10–12% of people living with HIV know their serostatus [9]. In Ethiopia most of the adult segment of the population (95%) does not know their HIV status [10]. And ART has been accessed by only 13% of those who need ART [11].

Currently, acceptability of provider initiated HIV testing and counseling (PITC) among TB patients varies from place to place. In Ethiopia the acceptability of PITC may be influenced by different associated factors like fear of stigma and discrimination associated with having TB and HIV, sociodemographic factors, knowledge related to TB/HIV, the general perception of HIV infection, and attitude towards PITC may affect the acceptance and knowledge of TB patients. PITC is one of the main intervention areas to fight against failure to meet TB control targets in areas where HIV prevalence was high, particularly, for those coinfected patients with TB and HIV.

Therefore, the aim of this study was to assess the acceptance of PITC and factors affecting PITC acceptance among TB patients. Furthermore, the results of this study help to design and practice appropriate interventions that decrease the burden of HIV on TB patient by enhancing the utilization of PITC service and are also important to improve treatment outcome by preventing other infectious diseases and decrease life loss of AIDS cases secondary to tuberculosis.

## 2. Materials and Methods 

An institution based, cross-sectional study was conducted from July to August 2010 among adult tuberculosis patients in the East Wollega administrative zone, Oromia regional state, western Ethiopia. It is located at 262 KMs from Jimma University and 331 KMs from Addis Ababa (the capital city). The expected population of the zone for 2005 was estimated to be 1,647,576. The zone was divided into 21 administrative districts. There were 2 hospitals, one zonal hospital and one district hospital, 15 health centers, 82 health stations, and 107 health posts; all were public [12]. All tuberculosis patients in selected health centers registered for directly observed treatment short course (DOTS) from October 8, 2009, to August 18, 2010, and TB cases encountered during the data collection period were included in the study and those TB patients below the age of 18 years and those critically ill patients are excluded from the study.

A single population proportion formula is used to calculate the total sample size of 406 and cluster sampling technique was used to select woredas based on their geographical location. The health center was selected from the total health centers using a simple random sampling technique. The number of participants was proportionally allocated to each health center. The identification number (ID number) of TB patients at each health center was written on a piece of paper and coiled and then the actual number of 406 study units was selected by using the lottery method. A structured questionnaire was used to obtain sociodemographic information (residence, ethnicity, sex, age, religion, marital status, educational status, occupation, and family size).

The study protocol was reviewed and approved by the Institutional Ethical Review Board of the Jimma University. The objective of the study was explained to the participants and verbal consent was obtained from each participant. Confidentiality of the result was kept by coding patient information. The data were checked for their completeness before entering for analysis; then all the data from study participants were imported into excel and analyzed by SPSS version 20 program software. Odds ratio, 95% confidence interval, and both bivariate and multivariate logistic regression were used to test the association between the outcome and independent variables.

## 3. Results

### 3.1. Sociodemographic

A total of 406 TB patients were included in the study and are with a 100% response rate. Of the total, 234 (57.6%) of the study participants were male with nearly 1.36 to 1 sex ratio. The mean age of the study populations was 35 years with 1.2 standard deviation (SD); more than half (68.2%) of the interviewed religions were Orthodox, followed by Protestant 120 (29.6%); 391 (96.3%) and 278 (68.5%) were identifying themselves as an Oromo ethnic group and live in urban areas, respectively. Marriage characteristics, educational status, family size, and monthly income per month were identified (Table 1).

### 3.2. Knowledge about TB/HIV/AIDS

Almost all study participants 404 (99.5%) believed that TB is a curable disease. 306 (75.4%) of the respondents believed that a person who lives with HIV/AIDS is at risk of acquiring tuberculosis 327 (80.1%). Likewise 359 (84.4%) participants agreed that controlling of HIV/AIDS is important in controlling process of TB infection. Study participants were able to identify the common means of HIV transmission and agreed that HIV/AIDS is not a curable disease. 87.9% of participants believed that they were at risk of getting a virus in the past, while the rest did not (Table 2). However, still there is a misconception on transmission of HIV/AIDS by mosquito bite accounting for 14.8% and wearing shared clothes (7.1%) with their friends.

### 3.3. Knowledge of Study Participants on Provider Initiated HIV Testing and Counseling (PITC) Services

**Figure 1:**
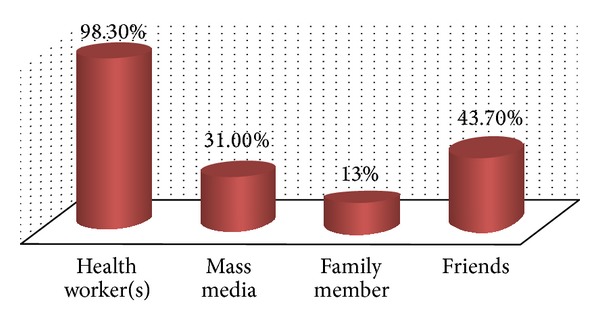
Source of information about PITC, among TB patients in East Wollega administrative zone at selected health centers, August 2010.

The majority of the study participants had heard about PITC before the time of data collection and the primary health workers were cited as a source of this information accounting for 98.3% followed by friends (43.7%) (Figure 1).

### 3.4. HIV Testing and Acceptance of Provider Initiated HIV Testing and Counseling Services

Of all the study participants 399 (98.2%) are initiated for HIV test by their TB treatment supervisor during TB treatment follow-up; of those, 369 (92.5%) TB patients accepted the initiation and 353 (95.7%) patients had undergone HIV test and received their test result. On the other hand, about 30 (7.5%) patients did not accept the initiation because of different reasons like waiting until TB treatment completion, unable to cope following positive result and the remaining 7 (1.8%) participants are not encouraged for PITC. 357 (87.9%) did not know their serostatus about HIV before the study period; of those 92 (25.5%) TB patients were willing to check their status for HIV in the past after advice was given. According to document review result the prevalence of HIV among TB patients who underwent HIV counseling and testing from October 8, 2009, to August 18, 2010, was 137 (33.7%).

### 3.5. Analysis of Factors Associated with Acceptance of PITC among TB Patients

In bivariate logistic regression analysis, each explanatory variable with the outcome variable (acceptance of PITC) was assessed for its association. Variables with *P* value >0.3 were not imported to multiple logistic regression for further analysis. Participants with formal education were 3.51 times more likely to accept PITC following their TB treatment supervisor initiation, as compared with who had not formal education (AOR = 3.51, 95% CI: 1.13–10.91). Similarly, those who had comprehensive knowledge about HIV/AIDS way of transmission were 3.22 times more likely to accept PITC than who had not (AOR = 3.22, 95% CI: 1.3–7.97). Who understand the importance of controlling HIV/AIDS helps in controlling of TB were 3.89 times more likely to accept PITC than those who do not know (AOR = 3.89, 95% CI: 1.54–9.83) (Table 3).

## 4. Discussion

Most of the TB patients were initiated for HIV test by their TB treatment supervisors 399 (98.7%) during TB treatment follow-up. Among those initiated 369 (92.5%) accepted PITC and of those 353 (95.7%) were tested and received their test result. In this study there is a high acceptance of PITC (92.5%) among TB patients. The study results that were not comparable with the previous studies conducted in Addis Ababa (capital city of Ethiopia) and Arba Minch (south nation, nationality people) among TB patients showed 66.6% and 73%, respectively [13, 14]. This difference may relate to the recent introduction of free life-saving antiretroviral therapy at selected health centers throughout Ethiopia. Similarly, this finding was comparable with studies done in India, Kenya, Cameroon, and Congo which showed 97%, 91.5%, 94.5%, and 96.25%, respectively [15, 16]. The reason for increment number of acceptances was that those patients who refuse to undergo an HIV test at the beginning of TB treatment may accept PITC initiation during TB treatment follow-up.

According to documents reviewed result, the prevalence of HIV among TB patients who undergo HIV test was 33.7%. This result is comparable with studies done in North West Ethiopia and South Ethiopia [17–19]. However, this finding is not in line with the Federal HIV/AIDS prevention and a control office report in March 2010 which accounts for 20% [17]; the reason for this might be that the increment of awareness and knowledge on TB-HIV infection and PITC services during TB treatment follow-up may increase the chance of positivity.

Patients who had self-perceived risk of contracting HIV/AIDS accept PITC as compared to not self-perceived risk patients (OR = 3.73 95%, CI: 1.6–8.73). This result was in agreement with a study done in Arba Minch hospital among TB patients that showed self-perceived risk patients were five times more willing to accept the initiation than non-self-perceived risk patients [20]. The possible explanation for this may be those patients who considered themselves at risk of getting HIV in the past are more interested to know their serostatus in order to benefit from the available treatment options related to HIV including provision of ART as compared to not self-perceived risk individuals (Table 3). Similarly, patients who had knowledge about provider initiated HIV testing and counseling service were more likely to accept PITC than unknowledgeable patients (OR = 8.09, 95%, CI: 1.83–35.67). This finding is in agreement with study done in North West Ethiopia [17].

Generally, in this study there is a high acceptance of PITC among TB patients following the initiation of TB treatment supervisor. This is important for the control, prevention, and treatment of TB-HIV coinfection strategies. Despite the high knowledge of TB/HIV, still there is a misconception about TB/HIV means of transmission. Therefore, information or health education about TB and HIV, particularly on way of transmission, prevention, and control, should be provided intensively by health extension workers during home-to-home visiting and TB treatment supervisors provide counseling regularly for all forms of TB patients.

## Figures and Tables

**Table 1:** Sociodemographic characteristics of tuberculosis patients in East Wollega administrative zone at selected health centers, August 2010 (*N* = 406).

Variables	Frequency	Percentage (%)
Residence		
Urban	278	(68.5)
Rural	128	(31.5)
Ethnic group		
Oromo	391	(96.3)
Amharic	13	(3.2)
Others*	2	(0.5)
Sex		
Male	234	(57.6)
Female	172	(42.4)
Age		
19–34	236	(58.1)
35–50	125	(30.8)
>50	45	(11.1)
Religion		
Orthodox	277	(68.2)
Protestants	120	(29.6)
Others**	9	(2.2)
Marital status		
Single	80	(19.7)
Married	257	(63.3)
Divorced/widowed/separated	69	(17.0)
Educational status		
Not formal education	230	(56.7)
Formal education	176	(43.3)
Occupational status		
Government employee	37	(9.1)
Farmer	130	(32.0)
House wife	113	(27.8)
Others***	126	(31.0)
Family size		
≤4	236	(58.1)
>4	170	(41.9)

Others*: Gurage; others**: Muslim; Catholic; others***: merchant, daily labor, student, and unemployed classified by using mean score.

**Table 2:** Knowledge related to TB/HIV/AIDS among tuberculosis patients in East Wollega administrative zone at selected health centers, August 2010, (*N* = 406).

TB/HIV/AID related questions	Frequency	(%)
Source of TB^#^		
From TB patients	402	(99.0)
Polluted air	381	(93.8)
Contaminated water	81	(20.0)
Lump of earth	93	(22.9)
Others*	8	(1.90)
Risk of acquiring TB^#^		
Poor people	328	(80.8)
Living with TB patient	380	(93.6)
Having HIV/AIDS	306	(75.4)
TB increased after the era of HIV/AIDS		
Increase	327	(88.4)
No difference	79	(11.6)
Controlling of HIV/AIDS is important in TB control		
Yes	359	(88.4)
No	47	(88.4)
Route of HIV transmission^#^		
Sexual contact	406	(100)
Mother to child	402	(99.0)
Transfusion of blood	404	(99.5)
Sharing of sharp materials with PLHIV	406	(100)
Blood contact	402	(99.0)
Mosquito bite	60	(14.8)
Share wearing clothes	29	(7.40)
Others**	3	(0.70)
Methods of HIV prevention^#^		
Limiting sex to one uninfected partner	404	(99.5)
Abstain from sexual intercourse	401	(98.8)
Use of condom during sexual intercourse	392	(96.6)
Comprehensive knowledge of HIV		
Knowledgeable	338	(83.3)
Not knowledgeable	68	(16.7)
Self-perceived risk of HIV infection		
No risk	48	(11.8)
Risk	358	(88.2)

^#^Multiple responses; others*: sexual intercourse, evil spirit; others**: shaking hand, sharing meal.

**Table 3:** Bivariate and multiple logistic regression analysis of factors associated with acceptance of PITC (*n* = 369).

Variables	Accepter	Not accepter	OR (95% CI)	AOR (95% CI)	*P* value
Educational status					**0.03**
Not formal education^R^	200	26	1.0	1.0	
Formal education	169	4	5.49 (1.88–16.05)	3.51 (1.13–10.91)	
Controlling of HIV could help in control of TB					**0.04**
No^R^	36	10	1.0	1.0	
Yes	333	20	4.63 (2.01–10.64)	3.89 (1.54–9.83)	
Comprehensive knowledge on HIV/AIDS					**0.011**
Not knowledgeable^R^	49	13	1.0	1.0	
Knowledgeable	320	17	4.99 (2.84–10.92)	3.22 (1.3–7.97)	
Self-perceived risk of contracting HIV					**0.028**
No risk^R^	38	9	1.0	1.0	
Risk	331	21	3.73 (1.6–8.73)	2.93 (1.12–7.66)	
Knowledge of PITC					**0.017**
Not knowledgeable^R^	5	3	1.0	1.0	
Knowledgeable	364	27	8.09 (1.83–35.67)	7.56 (1.14–40.35)	

^R^Reference category.
